# Zonulin as marker of pregnancy induced hypertension: a case control study

**DOI:** 10.1186/s40885-020-00139-x

**Published:** 2020-04-15

**Authors:** Ahmed Tijani Bawah, Henry Tornyi, Mohammed Mustapha Seini, Lincoln Toamsoma Ngambire, Francis Agyemang Yeboah

**Affiliations:** 1 Department of Medical Laboratory Sciences, University of Health and Allied Science, PMB 31, Ho, Ghana; 2 Department of Molecular Medicine, Kwame Nkrumah University Science and Technology, Kumasi, Ghana

**Keywords:** Zonulin, Pregnancy induced hypertension, Tight junctions

## Abstract

**Background:**

Zonulin has been implicated in many metabolic disorders including hypertension and obesity. However, there is insufficient information about the involvement of zonulin in pregnancy induced hypertension (PIH) which comprises preeclampsia (PE) and gestational hypertension (GH). This study was therefore aimed at finding the level of this biochemical marker of regulation of tight junctions among women with PIH.

**Methods:**

A total of 88 women with PIH and 60 age and body mass index (BMI) matched healthy pregnant women controls were enrolled. Blood pressure at 11–13 weeks and after 20 weeks of gestation, body mass index (BMI) in addition to serum Zonulin levels and lipid profile were compared between the groups. Student’s t-test was used for comparisons of the mean between the two groups. Correlation analyses were performed using Pearson’s correlation and binary logistic regression was used to evaluate the factors associated with PIH.

**Results:**

Zonulin level was significantly higher in the participants with PIH as compared to the normal pregnant controls 56.81 ± 7.72 ng/ml vs 40.4 ± 8.60 ng/ml *p* < 0.0001 and had strong positive correlation with PIH (OR = 1.805; CI1.139–1.275; p < 0.0001). However, the association between first trimester lipids and PIH was weak.

**Conclusion:**

The results showed a strong positive correlation between zonulin and PIH, thus changes in intestinal permeability occur in early stages of pregnancy and may be involved in the pathogenesis of PIH.

## Background

Recent evidence shows a relation between gut pathology and microbiome with hypertension in animal models. However, this association in humans is yet to be fully elucidated. The most important regulator of tight junctions (TJs) is human zonulin, a 47-kDa human protein that increases the permeability of the small intestine. It increases gut permeability by reversibly modulating the TJs, ensuring normal functions of the TJs as well as maintaining physiological processes in the intestines [1]. Furthermore, increased plasma level of zonulin has been shown to be a good marker of intestinal permeability [2, 3] and its secretion is triggered by gluten and bacteria [4]. Circulating level of zonulin had been associated with coronary heart disease [2]. Recent evidence point to the fact that increased permeability of gut epithelial barrier and inflammatory state is associated with blood pressure elevation [5]. However, there is insufficient information about zonulin’s participation in some important states of hypertension such PIH. PIH is divided into four groups of disorders. These are; chronic hypertension (CH), gestational hypertension (GH), preeclampsia and preeclampsia superimposed. PE (blood pressure ≥ 140/90 in association with proteinuria ≥300 mg in 24 h urine evidenced by the presence of vasoconstriction, metabolic changes, endothelial dysfunction, activation of coagulation cascade and an increase in inflammatory response) [6]. GH is defined as onset of hypertension (systolic blood pressure ≥ 140 mmHg and/or diastolic blood pressure ≥ 90 mmHg) after 20 weeks of gestation in the absence of proteinuria and usually associated with good maternal and fetal outcomes [7]. Current hypothesis of PE, an aspect of PIH attributes the pathophysiology to the placenta [8]. During the second trimester of pregnancy, abnormal placentation occurs due to remodeling of the spiral arteries, release of syncytiotrophoblast pro-inflammatory factors which are believed to cause systemic inflammatory response and clinical manifestations of PIH [9]. The actual mechanism of disturbed placentation is still unknown though the role of the proinflammatory factors have scientific plausibility.

Most chronic inflammatory diseases (CIDs) are associated with elevated zonulin levels including; autoimmune diseases [10], lung diseases [1], intestinal diseases [11], metabolic disorders [12] and heart diseases [2]. Due to the involvement of zonulin in many CIDs, including its role in blood pressure elevation and the fact that to date, no relationship between zonulin levels and PIH in Ghanaian community has been documented, we investigated the relationship between zonulin levels and hypertensive disorders of pregnancy among Ghanaians in Abor-Weme, a community in the Volta region of Ghana.

## Methods

This was a case-control study involving 148 participants (88 women with PIH and 60 women without PIH) at the Sacred Heart Hospital, Abor-Weme in the Volta Region of Ghana between January, 2017 and December, 2017. The participants were matched for age and BMI.

### Study population and criteria for selection

Those included in the study were pregnant women above 18 years with or without hypertension (cases and controls respectively). Pregnant women without dipstick proteinuria and whose blood pressure was < 140/90 mmHg were enrolled as controls while those with blood pressure ≥ 140/90 mmHg were enrolled as cases. Among the cases, those with concurrent proteinuria were diagnosed with PE while those without proteinuria were regarded as having GH. Those excluded were pregnant women with renal disease, diabetes, cancers, multiple pregnancy and pre-gestational hypertension.

### Anthropometric measurement

The participants wore light clothing and stood on a bathroom scale (Zhongshan Camry Electronic Co. Ltd., Guangdong, China) after they had removed their shoes; their weights were recorded to the nearest 0.1 kg. Height was measured without shoes with a stadiometer to the nearest 0.5 cm with the study participants standing upright and heels put together and the head in the horizontal plane. BMI was calculated as weight/height squared (Kg/m^2^).

### Blood pressure measurement

Blood pressure was measured using a mercury sphygmomanometer and stethoscope. Measurements were taken from the left upper arm after the participants had rested for at least 5 min in accordance with the guidelines of the American Heart Association [13].

Triplicate measurements were taken with at least 5 min waiting period between measurements and the mean blood pressure was recorded to the nearest 2.0 mmHg.

### Collection and preservation of samples

After 20th week of gestation, each participant was provided with clean dry, wide mouth, leak proof container to collect about 5 ml of urine sample. During that same period, five milliliter of blood samples were also taken between 7:00 am and 8:00 am after an overnight fast of between 10 to 14 h and put into serum separator tubes and immediately put on ice packs. Serum samples were separated within 1 h and then stored in several aliquots at -80 °C for subsequent biochemical analysis.

### Biochemical and urine analysis

Urine strips were inserted into the urine samples up to the test area for less than 2 s. The edge of the strips were drawn along the brims of the vessels to remove excess urine, making sure the tests areas did not touch the vessels. The strips were held vertical and the tips tapped on absorbent papers to remove any remaining urine [14]. The urine strips were held horizontally and compared with the color charts on the vial label under bright light. The amount of protein was then determined using the intensity of the blue green color which was proportional to concentration of protein in the urine. Proteinuria was defined as the presence of urinary protein with concentration of at least “+” [15]. The Lipid Profile was determined using the Selectra Pro S (Vital Scientific B.V. Van Rensselaerwweg 4, NL 6956AV Spankeren, The Netherlands) automated chemistry analyzer using the procedure outlined for the equipment while serum zonulin levels were determined in duplicates using human zonulin ELIS AKit (Immundiagnostik AG, Bensheim, Germany).

### Study variables and outcome measurement

Every pregnant woman in this hospital is screened for PE after the 20th week of gestation. The primary outcome was PIH occurrence (yes/no), which was determined according to the PIH diagnosis criteria. Blood pressure measurement was done after 20 weeks of pregnancy and urine protein determined during the same period using the dip-stick qualitative/semi-quantitative method (Urit Medical Electronic Co., Ltd., Guangxi, People’s Republic of China). Diagnosis of PE was performed by qualified Obstetrician/Gynecologist based on systolic and diastolic blood pressure of 140 mmHg or more and 90 mmHg or more respectively (or both) on two occasions at least 4 h apart accompanied by proteinuria of + or more. Those with systolic blood pressure of 140 mmHg or more and diastolic blood pressure of 90 mmHg or more or both without concurrent proteinuria were diagnosed with GH.

### Statistical analysis

All data analyses were performed using the SPSS software (version 22.0 systat, Inc. Germany) and GraphPad Prism, (version 5.0, San Diego California, USA). Data was presented as mean ± SD. In all the statistical analysis, a value of *p* < 0.05 was considered to be significant and at a 95% confidence interval. Pearson correlation was used to evaluate correlation of biochemical and anthropometric parameters and PIH. Logistic regression was used to evaluate the effect of blood pressure at time of enrollment and biochemical parameters on the prediction of PIH.

## Results

A total of 148 participants were recruited into the study comprising 60 women with normal pregnancies and 88 pregnant women with PIH. Among those with PIH, 30 of them had PE and 58 had GH. There were no statistically significant differences in the age and BMI between the cases and control (Table 1). The first trimester systolic and diastolic blood pressure (BP) of both controls and cases were normal (< 140/90 mmHg), however, there was significant difference between them. The mean systolic BP for the cases and the controls was 114.32 ± 8.41 mmHg and 110.83 ± 9.44 mmHg respectively, *p* = 0.020 while the diastolic BP was 73.52 ± 8.98 mmHg and 70.18 ± 7.05 mmHg respectively for the cases and the controls, *p* = 0.017 (Table 1). The gestational age at which participants delivered was also significantly different between the cases and the controls (37.91 ± 2.84 weeks vs 38.93 ± 1.33 weeks respectively, *p* = 0.010). Comparison of the biochemical parameters between the participants revealed no significant difference in the TG and LDL but there were significant differences in the TC, HDL and VLDL.

**Table 1:** Comparison of anthropometric and biochemical markers among the participants

Variable	Normal Pregnancy (*N* = 60)	PIH (*N* = 88)	*p* values
AGE (Years)	27.98 ± 6.43	29.10 ± 5.32	0.250
BMI (Kg/m^2^)	27.86 ± 4.19	28.96 ± 4.121	0.116
Blood pressure at time of enrollment			
SBP(mmHg)	110.83 ± 9.44	114.32 ± 8.41	0.020
DBP(mmHg)	70.18 ± 7.05	73.52 ± 8.98	0.017
Blood pressure at time of diagnosis PIH			
SBP (mmHg)	118.33 ± 9.23	150.23 ± 14.06	> 0.001
DBP (mmHg)	75.33 ± 9.47	96.14 ± 11.98	> 0.001
GA (weeks)	38.93 ± 1.33	37.91 ± 2.84	0.010
Zonulin (ng/ml)	40.4 ± 8.60	56.81 ± 7.72	> 0.001
TG (mmol/L)	2.08 ± 0.92	2.35 ± 0.78	0.061
TC (mmol/L)	6.42 ± 2.03	7.11 ± 1.90	0.036
HDL-C (mmol/L)	1.24 ± 0.92	0.91 ± 0.83	0.026
LDL-C (mmol/L)	4.60 ± 1.91	5.21 ± 1.89	0.058
VLDL-C (mmol/L)	0.83 ± 0.41	0.96 ± 0.34	0.036

*BMI* Body mass index, *PIH* Pregnancy induced hypertension, *GA* Gestational age, *TG* Triglycerides, *TC* Total cholesterol, *HDL-C* High density lipoprotein cholesterol, *LDL-C* Low density lipoprotein cholesterol, VLDL-C cholesterol. Data is presented as mean ± SD.

Zonulin level was significantly higher in the participants with PIH as compared to the normal pregnant controls 56.81 ± 7.72 ng/ml vs 40.4 ± 8.60 ng/ml *p* < 0.001. Comparison of clinical and biochemical parameters between those with PE and GH revealed no statistically significant difference in all the parameters with the exception of systolic BP at time of enrollment (116.57 ± 7.65 vs 112.83 ± 8.63) mmHg, respectively, *p* = 0.040 (Table 2).

**Table 2:** Comparison of anthropometric and biochemical markers among the participants with PE and GH

Variable	PE (*N* = 30)	GH (*N* = 60)	p values
AGE (Years)	28.60 ± 3.99	29.43 ± 6.05	0.474
BMI (Kg/m^2^)	29.46 ± 4.00	28.63 ± 4.20	0.353
Blood pressure at time of enrollment			
SBP(mmHg)	116.57 ± 7.65	112.83 ± 8.63	0.040
DBP(mmHg)	75.71 ± 6.55	72.08 ± 10.07	0.062
Zonulin (ng/ml)	57.08 ± 7.64	56.634 ± 7.84	0.796
TG (mmol/L)	2.4543.68337	2.23 ± 0.84	0.306
TC (mmol/L)	7.40 ± 1.56	6.9151 ± 2.07	0.234
HDL-C (mmol/L)	0.85 ± 0.79	0.95 ± 0.86	0.580
LDL-C (mmol/L)	5.41 ± 1.8346	5.08 ± 1.92	0.416
VLDL-C (mmol/L)	1.01 ± 0.29	0.93 0.37	0.290

*BMI* Body mass index, *TG* Triglycerides, *TC* Total cholesterol, *HDL-C* High density lipoprotein cholesterol, *LDL-C* Low density lipoprotein cholesterol, VLDL-C cholesterol. Data is presented as mean ± SD.

Correlation of the anthropometric and biochemical parameters and PIH revealed weak correlation between first trimester systolic blood pressure and diastolic blood pressure and PIH. There were also weak positive correlations between PIH and some lipid fractions; TC, HDL and VLDL. There was a strong positive correlation between serum zonulin level and PIH (r = 0.708, *p* < 0.0001) (Table 3).

**Table 3:** Correlation of anthropometric, biochemical parameters of participants and Pregnancy induced Hypertension

		DBP	SBP	ZONULIN	TG	TC	HDL	LDL	VLDL	PIH
DBPATB	P	0.648	0.218	0.222	0.794	0.015	0.706	0.029	0.658	0.116
	R	1	0.250**	0.184*	0.106	0.155	−0.242**	0.147	0.081	0.196*
SBPATB	P		0.002	0.025	0.198	0.06	0.003	0.075	0.327	0.017
	R		1	0.118	0.074	0.189*	−0.037	0.111	0.109	0.191*
ZONULIN	R			1	0.127	0.163*	−0.109	0.161	0.138	0.708**
	P				0.124	0.047	0.188	0.051	0.094	< 0.0001
TG	R				1	.573**	−0.461**	0.288**	0.954**	0.155
	P					< 0.0001	< 0.0001	< 0.0001	< 0.0001	0.061
TC	R					1	−0.487**	0.760**	0.598**	0.172*
	P						< 0.0001	< 0.0001	< 0.0001	0.036
HDL	R						1	−0.498**	−0.406**	− 0.183*
	P							< 0.0001	< 0.0001	0.026
LDL	R							1	0.207*	0.156
	P								0.012	0.058
VLDL	R								1	0.173*
	P									0.036
PIH	R									1
	P									

*DBP* Diastolic blood pressure at time of enrollment, *SBP* Systolic blood pressure at time of enrollment, *DBPATB* Diastolic blood pressure at booking, *SBPATB* Systolic blood pressure at booking, *TG* Triglycerides, *TC* Total cholesterol, *HDL* High density lipoprotein cholesterol, *LDL* Low density lipoprotein cholesterol, *VLDL* Very low density lipoprotein cholesterol. *PIH* Pregnancy induced hypertension.

**Correlation is significant at the 0.01 level (2-tailed). * Correlation is significant at the 0.05 level (2-tailed)

After controlling for factors associated with PIH through binary logistic regression, there were variations in the *p* values for the biochemical and clinical characteristics but Zonulin was stronglyassociated with PIH (OR = 1.805; 95% CI =1.139–1.275; *p* < 0.001) showing that women with elevated plasma zonulin levels were two times more likely to develop either preeclampsia or gestational hypertension as compared to those with normal plasma zonulin level (Table 4).

**Table 4:** Multivariate logistic regression of factors and biochemical markers associated with PIH

Variable	Regression coefficient (β)	OR (95% CI)	*P value*
Age (Years)	0.028	1.028 (0.937–1.128)	0.556
BMI (kg/m^2^)	0.053	1.055 (0.925–1.202)	0.424
DBPATB (mmHg)	0.023	1.023 (0.954–1.096)	0.524
SBPATB (mmHg)	0.037	1.038 (0.979–1.100	0. 209
Zonulin (ng/ml)	0.186	1.805 (1.139–1.275)	0.001
TG (mmol/L)	−1.087	0.337 (0.034–3.303)	0.351
TC (mmol/L)	−0.224	0.799 (0.436–1.465)	0.468
HDL(mmol/L)	0–.555	0.574 (0.275–1.195)	0.138
LDL (mmol/L)	0.170	1.185-(0.665–2.111)	0.564
VLDL (mmol/L)	3.114	22.506 (0.068–7436.610)	0.293

**Fig. 1:**
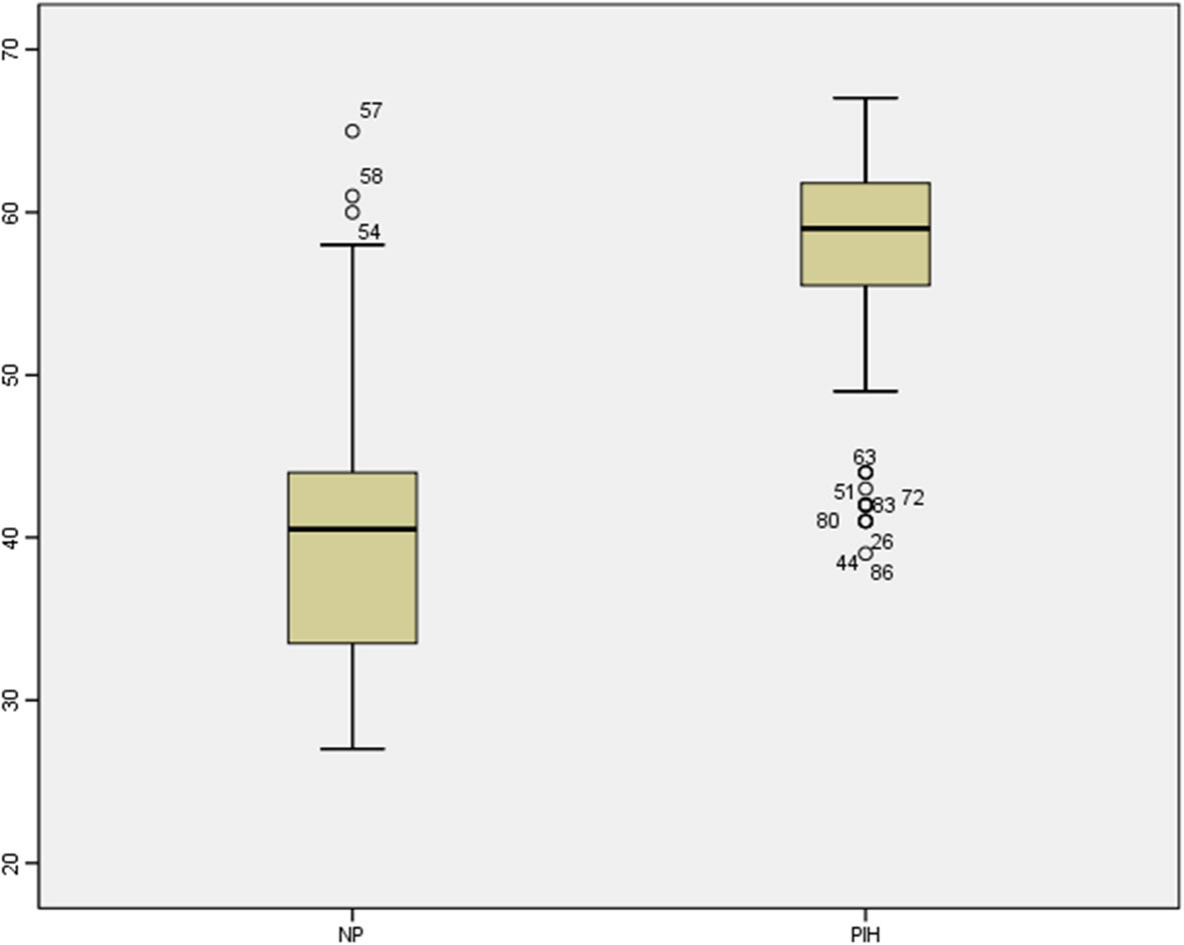
Box-Whisker plop of Zonulin NP = normal pregnancy, PIH = pregnancy induced hypertension

**Fig. 2:**
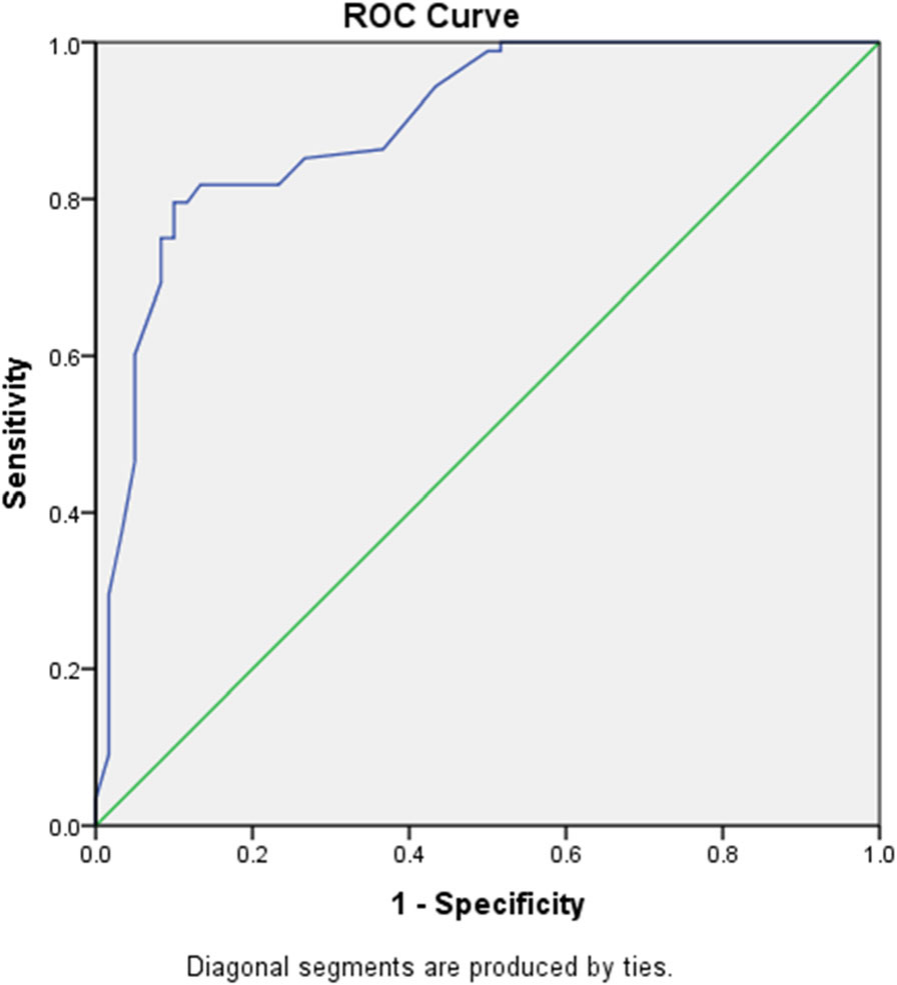
Receiver operating characteristic curve for zonulin and PIH. AUC = 0.8943, sensitivity = 75.0%,, specificity = 91.7%, likelihood ratio = 9, Cut off > 56.5 ng/ml

The box-whisker plot of zonulin in normal pregnancy and PIH showed significantly higher zonulin in PIH compared to the normal pregnancy (Fig. 1). The area under the ROC curve (AUC) (Fig. 2) showed that zonulin had high accuracy of predicting PIH with AUC of 0.894, sensitivity, specificity and likelihood ratio of 75.0, 91.7% and 9 respectively at a cut off value of > 56.5 ng/ml.

## Discussion

We assessed the association between elevated plasma zonulin and pregnancy induced hypertension. Our study showed significantly higher serum zonulin in women with pregnancy induced hypertension as compared to those with normal pregnancy. There was no significant change in zonulin level after stratifying the data into those with preeclampsia and those with gestational hypertension. There was also strong correlation between PIH and elevated serum zonulin level. This finding is similar to a report which stated that markers of increased gut permeability, especially zonulin, predicted systolic blood pressure and that gut barrier dysfunction and gut microbiome composition are directly linked with hypertension in humans [16]. In another study, the researchers reported that PE diagnosed during the third trimester of pregnancy was associated with a disrupted gut microbiota composition compared with those without PE and that serum zonulin was higher in the women with PE compared those with uncomplicated pregnancies though the difference was not statistically significant [17].

The association between zonulin levels and PIH is in accordance with previous studies which have shown that zonulin positively correlates with systolic blood pressure [18]. In another study it was reported that patients with hypertension did not have higher zonulin level compared with controls none the less they demonstrated that there was correlation between zonulin and systolic blood pressure [19]. Diastolic blood pressure has also been shown to be associated with higher zonulin level, overweight, hyperlidemia and fasting blood glucose [20]. The possible mechanism by which zonulin may be involved in PIH is that it could lead to activation of neurohumoral systems involving renin-angiotensin-aldosterone system, sympathetic nervous system and antidiuretic hormone. When there is activation of these systems, plasma volume will increase leading to hypertension [21]. Furthermore, elevated plasma zonulin affect the intestinal barrier and when the intestinal barrier is bridged, infectious agents and dietary antigens to mucosal immune elements gain access to the body which may lead to increase in immune reactions and induce inflammation leading to PIH. Elevated zonulin may also lead to changes in gut junction proteins resulting in permeability changes, and dysbacteriosis. Gut dysbacteriosis and increased lactate producing bacteria populations have been shown to increase in hypertensive rats [22]. Neurotransmitters like dopamine [23] histamine [24] and serotonin [25] which may potentially be involved in blood pressure regulation are also produced by these gut bacteria and so when there is dysbiosis which is usually associated with elevated zonulin during pregnancy, hypertensive disorders of pregnancy may occur.

Based on this result, it may be deduced that changes in intestinal permeability occur in early stages of hypertensive disorders of pregnancy before progressing to preeclampsia and eclampsia. Large-scale prospective research on the underlying relationship between changes in intestinal permeability which is demonstrated by elevated zonulin level and PIH may be necessary. Furthermore, gaining a sufficient understanding of the pathogenesis of pregnancy induced hypertension associated with increased intestinal permeability and finding ways to avert the development and worsening of pregnancy associated hypertension may be important.

This study did not demonstrate significant differences in triglycerides and low density lipoprotein cholesterol between women with PIH and those with normal pregnancies. This is contrary to a study done in Brazil in which significant differences in triglyceride (TG)-rich proteins – very low-density lipoprotein 1 and low-density lipoprotein III were reported between women with PE and normal pregnant women [26]. Our study did not measure those specific TG-rich proteins, and this could also be the reason for the differences in our findings and what they reported. Our study however showed significant differences in the total cholesterol (TC), high density lipoprotein cholesterol (HDL-C) and very low density lipoprotein cholesterol (VLDL-C) between women with PIH and the normotensive pregnant women. This is in consonance with a previous study which concluded that human pregnancy was associated with an ‘atherogenic’ lipid profile that is further exacerbated in preeclampsia and that this profile may be a potential contributor to endothelial cell dysfunction [27]. We did not find any significant difference in lipid profile parameters between women with PE and GH.

### Study limitations

Limitations of the present study were the limited sample size and lack of nutritional data. Foods like leafy greens, berries and red beets are known to have blood pressure lowering effects. Leafy greens like spinach and klae have high potassium levels. Potassium helps the kidneys to get rid of more sodium through the urine and consequently lowers the blood pressure. The small sample size may be insufficient in drawing conclusion on the relationship between zonulin and PIH and also the nutritional status and medications could be not controlled in multivariate analysis.

## Conclusions

Plasma zonulin concentration in pregnancy induced hypertensive women was significantly elevated but there was no significant difference in plasma zonulin between those with preeclampsia and those with gestational hypertension. There was also strong correlation between elevated zonulin and pregnancy induced hypertension.

## Data Availability

The datasets used and/or analyzed during the current study are available from the corresponding author on reasonable request.

## Abbreviations

BMI: Body mass index
CID: Chronic inflammatory disease
DBP: Diastolic blood pressure
DBPATB: Diastolic blood pressure at booking
GH: Gestational hypertension
HDL: High density lipoprotein cholesterol
LDL: Low density lipoprotein cholesterol
PE: Preeclampsia
PIH: Pregnancy induced hypertension
SBP: Systolic blood pressure
SBPATB: Systolic blood pressure at booking
TC: Total cholesterol
TG: Triglycerides
VLDL: Very low density lipoprotein cholesterol

## References

[1] Fasano A (2011). Zonulin and its regulation of intestinal barrier function: the biological door to inflammation, autoimmunity, and cancer. Physiol Rev.

[2] Li C, Gao M, Zhang W, Chen C, Zhou F, Hu Z, Zeng C (2016). Zonulin regulates intestinal permeability and facilitates enteric bacteria permeation in coronary artery disease. Sci Rep.

[3] Mokkala K, Pellonperä O, Röytiö H, Pussinen P, Rönnemaa T, Laitinen K (2017). Increased intestinal permeability, measured by serum zonulin, is associated with metabolic risk markers in overweight pregnant women. Metabolism.

[4] Fasano A (2012). Zonulin, regulation of tight junctions, and autoimmune diseases. Ann N Y Acad Sci.

[5] Santisteban MM, Qi Y, Zubcevic J, Kim S, Yang T, Shenoy V, Cole-Jeffrey CT, Lobaton GO, Stewart DC, Rubiano A, Simmons CS, Garcia-Pereira F, Johnson RD, Pepine CJ, Raizada MK (2017). Hypertension-linked pathophysiological alterations in the gut. Circ Res.

[6] Khosravi S, Dabiran S, Lotfi M, Asnavandy M (2014). Study of the prevalence of hypertension and complications of hypertensive disorders in pregnancy. Open J Preven Med.

[7] Helewa ME, Burrows RF, Smith J, Williams K, Brain P, Rabkin SW (1997). Report of the Canadian Hypertension Society Consensus Conference: 1. Definitions, evaluation and classification of hypertensive disorders in pregnancy. CMAJ.

[8] Redman CWG (2011). Preeclampsia: a multi-stress disorder. La Revue de medicine interne.

[9] Redman CW, Sargent IL, Staff AC (2014). IFPA Senior Award Lecture: making sense of pre-eclampsia–two placental causes of preeclampsia?. Placenta.

[10] Smecuol E, Sugai E, Niveloni S, Vázquez H, Pedreira S, Mazure R, Moreno ML, Label M, Mauriño E, Fasano A, Meddings J, Bai JC (2005). Permeability, zonulin production, and enteropathy in dermatitis herpetiformis. Clin Gastroenterol Hepatol.

[11] Ling X, Linglong P, Weixia D, Hong W (2016). Protective effects of bifido bacterium on intestinal barrier function in LPS-induced enterocyte barrier injury of Caco-2 monolayers and in a rat NEC model. PLoS One.

[12] Moreno-Navarrete JM, Sabater M, Ortega F, Ricart W, Fernández-Real JM (2012). Circulating zonulin, a marker of intestinal permeability, is increased in association with obesity-associated insulin resistance. PloS One.

[13] Kirkendall WM, Burton AC, Epstein FH, Freis ED (1967). Recommendations for human blood pressure determination by sphygmomanometers. Circulation.

[14] Yeboah FA, Ngala RA, Bawah AT, Asare-Anane H, Alidu H, Hamid AM, Wumbee JDK (2017). Adiposity and hyperleptinemia during the first trimester among pregnant women with preeclampsia. Int J Womens Health.

[15] American College of Obstetricians and Gynecologists (2013). Hypertension in pregnancy. Report of the American College of Obstetricians and Gynecologists’ task force on hypertension in pregnancy. Obstet Gynecol.

[16] Kim S, Goel R, Kumar A, Qi Y, Lobaton G, Hosaka K, Mohammed M, Handberg EM, Richards EM, Pepine CJ, Raizada MK (2017). Plasma Zonulin, Along With a Unique Gut Microbiome Profile, Are Potential Predictors of Systolic Blood Pressure in Humans. Hypertension.

[17] Lv LJ, Li SH, Li SC, Zhong ZC, Duan HL, Tian C, Li H, He W, Chen MC, He TW, Wang YN, Zhou X, Yao L, Yin AH (2019). Early-onset preeclampsia is associated with gut microbial alterations in antepartum and postpartum women. Front Cell Infect Microbiol.

[18] Zak-Gołąb A, Kocełak P, Aptekorz M, Zientara M, Juszczyk L, Martirosian G, Chudek J, Olszanecka-Glinianowicz M (2013). Gut microbiota, microinflammation, metabolic profile, and zonulin concentration in obese and normal weight subjects. Int J Endocrinol.

[19] Malyszko J, Koc-Zorawska E, Levin-Iaina N, Malyszko J. Zonulin, iron status, and anemia in kidney transplant recipients: are they related? In: Transplantation proceedings: 2014. Elsevier: 2644–2646. 10.1016/j.transproceed.2014.09.018 25380885

[20] Ohlsson B, Orho-Melander M, Nilsson PM (2017). Higher levels of serum zonulin may rather be associated with increased risk of obesity and hyperlipidemia, than with gastrointestinal symptoms or disease manifestations. Int J Mol Sci.

[21] Makharia GK (2014). Intestinal permeabililty in portal hypertension: Still a dilemma. Trop Gastro Enterol.

[22] Yang T, Santisteban MM, Rodriguez V, Li E, Ahmari N, Carvajal JM, Zadeh M, Gong M, Qi Y, Zubcevic J, Sahay B, Pepine CJ, Raizada MK, Mohamadzadeh M (2015). Gut dysbiosis is linked to hypertension. Hypertension.

[23] Özogul F (2011). Effects of specific lactic acid bacteria species on biogenic amine production by foodborne pathogen. Int J Food Sci Tech.

[24] Rossi F, Gardini F, Rizzotti L, La Gioia F, Tabanelli G, Torriani S (2011). Quantitative analysis of histidine decarboxylase gene (hdcA) transcription and histamine production by Streptococcus thermophilus PRI60 under conditions relevant to cheese making. Appl Environ Microbiol.

[25] Lyte M (2011). Probiotics function mechanistically as delivery vehicles for neuroactive compounds: microbial endocrinology in the design and use of probiotics. Bioessays.

[26] Lima VJ, Andrade CR, Ruschi GE, Sass N (2011). Serum lipid levels in pregnancies complicated by preeclampsia. Sao Paulo Med J.

[27] Belo L, Caslake M, Gaffney D, Santos-Silva A, Pereira-Leite L, Quintanilha A, Rebelo I (2002). Changes in LDL size and HDL concentration in normal and preeclamptic pregnancies. Atherosclerosis.

